# Association of Glycated Proteins with Inflammatory Proteins and Periodontal Disease Parameters

**DOI:** 10.1155/2020/6450742

**Published:** 2020-01-10

**Authors:** Jeneen Panezai, Mohammad Altamash, Per-Erik Engstrӧm, Anders Larsson

**Affiliations:** ^1^Altamash Institute of Dental Medicine, Department of Periodontology, Karachi, Pakistan; ^2^Karolinska Institutet, Department of Dental Medicine, Division of Oral Diseases, Section of Periodontology, Huddinge, Sweden; ^3^Uppsala University, Department of Medical Sciences, Uppsala, Sweden

## Abstract

Periodontitis is a chronic inflammatory condition that may contribute to diabetogenesis. The aim was to investigate the levels of glycated proteins and their correlation with periodontal and systemic inflammation. Fifty-one patients with periodontitis and 20 healthy subjects underwent probing pocket depth (PPD) measurements. PPD total and PPD disease with and without tooth adjustment were used as continuous indices. Marginal bone loss (MBL) for mandibular premolars and molars was measured digitally. Body mass index (BMI) and waist circumference (WC) were also analyzed. Glycated hemoglobin (HbA1c) and fructosamine (FrAm) levels were measured in all subjects. A multiplex proximity extension assay (PEA) was used to analyze the serum samples for simultaneous measurement of 92 proteins. Both HbA1c and FrAm inversely correlated with IL-10, FGF-21, MCP-1, and TNF beta amongst 16 proteins. HbA1c correlated directly with OPG. Parameters of disease severity were consistently significant for HbA1c. Adjusted PPD total and number of missing teeth were increased in diabetes whereas levels of RANKL and RANKL to OPG ratio were the highest in nondiabetic periodontitis patients. Hyperglycemic conditions in periodontitis patients are associated with reduced levels of anti-inflammatory proteins as well as dysregulated bone resorption.

## 1. Introduction

Periodontitis is a chronic inflammatory disease which entails destruction of the bone and connective tissues surrounding the tooth [1]. It is frequently found with an attendant metabolic comorbidity better known as type 2 diabetes [2]. This may be due to an intricately balanced relationship found between the immune and metabolic response systems. In a healthy state, compensatory and adaptive measures keep this balance in check. However, conditions of sustained exposure to inflammation may cause disruption of the metabolic function [3]. Metabolic response in terms of chronic hyperglycemia adds burden to the quality of life with an impact on periodontal health and disease severity [4].

Evaluation of glycemic conditions prevailing over longer periods can be done by the use of the glycated proteins, glycated hemoglobin (HbA1c), and fructosamine (FrAm). HbA1c is glycated hemoglobin in which glucose is attached to the N-terminal valine residue of the beta chain of hemoglobin A (HbA). The extent of hemoglobin glycation is influenced by the concentration of glucose in the blood. Based on the life span of erythrocytes (~120 days), HbA1c, therefore, reflects the mean glucose concentration over the preceding 8–12 week period [5]. HbA1c is a validated and reliable marker for determining hyperglycemia and predicting complications related to it [6]. However, HbA1C measurement can be affected in cases of red blood cell disorders, and therefore, FrAm becomes useful [7]. In blood, FrAm is predominantly a measure of glycated albumin since albumin accounts for 60-70% of total serum proteins; however, other circulating proteins such as glycated globulins and lipoproteins may contribute to the total concentration [8]. Due to increased protein turnover, FrAm reflects average glucose levels during the preceding 1 to 3 weeks, therefore, providing an earlier indication of hyperglycemia [9]. We hypothesize that periodontitis disrupts glycemic control through inflammation-induced hyperglycemia.

The aim of this study was to determine the serum levels of glycated proteins and their relationship with periodontal parameters and inflammatory proteins in periodontitis patients.

## 2. Material and Methods

### 2.1. Patients

This cross-sectional study was performed at the Department of Periodontology, the Altamash Institute of Dental Medicine in Karachi, Pakistan. Informed consent was obtained from willing participants after having received information about the study. A detailed questionnaire was used to acquire information pertaining to hospitalization history and the presence of self-reported chronic diseases including type 2 diabetes. Information regarding smoking habits, use of medication, oral hygiene status, and past dental history was also recorded. The characteristics of patients and controls are provided in Table 1. All controls had clinically healthy periodontium and no systemic disease. The exclusion criteria were as follows: a history of treatment for periodontal disease during the last six months and/or treatment with antibiotics in the last three months.

### 2.2. Clinical Examination

Bleeding on probing (BOP) was recorded, followed by measurement of probing pocket depth (PPD) in all teeth except for third molars. BOP was recorded as the presence or absence of local bleeding within 30 seconds of probing. Periodontitis was defined as three different sites or more with PPD of ≥5 mm using a periodontal probe (Hu-Friedy Manufacturing, Chicago, IL, USA). The scores were recorded for four sites per tooth (distobuccal, midbuccal, mesiobuccal, and lingual/palatal) which, in a full dentition of 28, would give 112 sites. The number of sites with BOP was recorded as a mean percentage, while the number of sites with shallow (3-<5 mm) and deep pockets (≥5 mm) was noted numerically. Additionally, a novel *Σ*PPD total index was calculated by adding the probing pocket depth measurements from measured sites to give a total aggregate whose value is the sum of each of the PPD site measurement in millimeters. *Σ*PPD disease was calculated by adding the probing pocket depths measuring ≥5 mm. Adjustment was further done by dividing the total value by the number of teeth probed. All measurements were carried out by a single examiner (JP).

### 2.3. Radiographic Measurement

Digital panoramic radiographs were taken using a digital extra oral tomography machine (Sirona Orthophos 3, Dentsply Sirona, Germany). The digital radiographs were viewed on a computer screen allowing for digital measurements to be made using SIDEXIS software [10]. One pixel was equal to 0.09 mm. Marginal bone loss (MBL) was defined as a measurement of the vertical distance from the cement-enamel junction (CEJ) to the most apical portion of the marginal bone. MBL was analyzed for mandibular premolars and molars (excluding third molars). An average value for MBL per tooth was calculated after recording two subsequent readings of mesial and distal sides each on digital radiographs. The measurements were added to give a total arithmetic sum of MBL per individual denoted by *Σ*MBL. Tooth adjustment was done by dividing *Σ*MBL with the number of teeth measured.

### 2.4. HbA1c

Glycated hemoglobin levels were determined for all subjects. Four milliliters of whole blood was collected in BD Vacutainer™ spray-coated EDTA tubes (lavender top, Becton, Dickinson, Franklin Lakes, NJ, USA) for determination of HbA1c. The samples were submitted on the same day for HbA1c analyses in Karachi Laboratory Diagnostic Centre, Karachi, Pakistan. Hemoglobin A1c was analyzed using the ion-exchange high-performance liquid chromatography system Bio-Rad D-10 Hemoglobin Testing System (Bio-Rad Laboratories, Hercules, CA, USA). The HbA1c values are standardized according to the NGSP system [11].

### 2.5. Fructosamine

Fructosamine was analyzed on a BS380 analyzer (Mindray, Shenzhen, China) using reagents (04537939190) from Roche Diagnostics (Mannheim, Germany). Precimat fructosamine (REF 11098993, Roche Diagnostics) was used as a calibrator. The settings used were endpoint method with blank reading at position 33-35 and final reading at position 56-59, wavelength (sub/main) 700/546, 10 *μ*L sample volume, 150 *μ*L R1, and 54 *μ*L R2. The total coefficient of variation (CV) for the fructosamine method was corresponding total CVs that were 1.98% at 275 *μ*mol/L and 1.65% at 515 *μ*mol/L.

### 2.6. Proximity Extension Assay (PEA)

**Figure 1:**
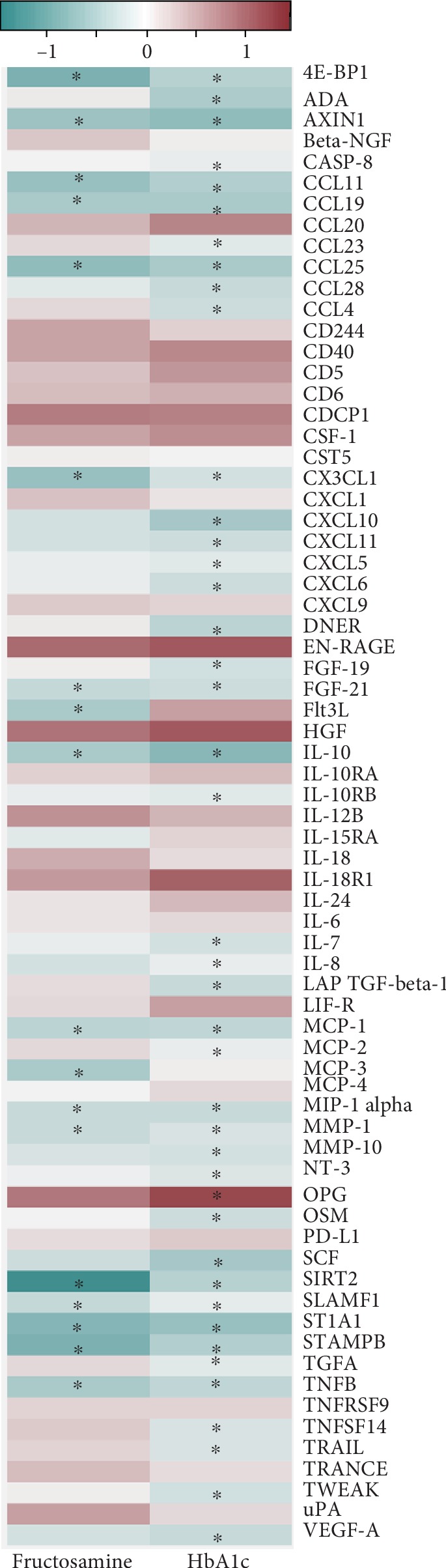
A correlation heat map of 70 inflammatory proteins associated with fructosamine and HbA1C in all study subjects (periodontitis = 38 + H = 12, *n* = 50). Protein intensities are displayed as colors ranging from green (negative) to maroon (positive) as shown in the key. Spearman rho coefficient *r* was calculated to determine the correlation. Correlations with adjusted *P* values ≤ 0.05 are marked with an asterisk.

A subset of serum samples (38 periodontitis patients and 12 controls) was used for conducting PEA using Proseek Multiplex Inflammation I (Olink Bioscience, Uppsala, Sweden), according to the manufacturer's instructions. The samples were collected in BD Vacutainer™ plastic blood collection tubes (4 mL) without any additives. Blood was allowed to coagulate, and tubes were then centrifuged at 1790 x *g* for 10 minutes. The serum was removed and transferred to 2 mL storage tubes and kept frozen at -22°C until the time of analyses. The panel simultaneously measures 92 biomarkers, as a homogeneous assay, in a 96-well microtiter plate format (http://www.olink.com/products/inflammation/). The samples were assayed as singletons. Methods pertaining to protein arrays, quality control, and data normalization have been discussed in detail in our previous publications [12, 13]. Calibrator curves for correlating the normalized protein expression (NPX) values with actual concentrations can also be found in Olink's website (http://www.olink.com/proseek-multiplex/inflammation/biomarkers/). The assay was performed blinded without knowledge of clinical data. Proteins with detectable levels in at least 50% of the samples were used for analytical purpose. This criterion was met by 70 out of 92 proteins (Figure 1).

### 2.7. Body Mass Index (BMI) and Waist Circumference (WC)

Anthropometric index measurements included weight (with the precision of 0.1 kg), height, and WC (at midpoint level between the inferior margin of the last rib and the iliac crest measured horizontally using a tape). BMI was calculated by using the following formula:

(1) $$\begin{array}{c} \frac{\text{Weight }\left(\text{kg}\right)}{{\left(\text{Height }\left(\text{m}\right)\right)}^{2}}. \end{array}$$

### 2.8. Classification Criteria for Diabetes

According to the guidelines of ADA, the diagnostic criteria applied in order to classify patients according to their glycemic status are shown in Table 2 [14]. Subjects who did not answer “yes” to having type 2 diabetes on the questionnaire but their HbA1c met the American Diabetes Association (ADA) cutoff for type 2 diabetes (HbA1c ≥ 6.5%) were classified as undiagnosed diabetes subjects.

### 2.9. Ethical Approval

The project and its protocols were approved by the Ethical Committee at the Altamash institute of Dental Medicine, Karachi, Pakistan (2012-09-26, 2016-09-30), and the Regional Ethical Review Board in Stockholm, Sweden (2016/296-31/1). The project work was conducted in accordance with the Declaration of Helsinki.

### 2.10. Statistical Analyses

All statistical analyses were performed using a software program SPSS version 21.0 (SPSS Inc., Chicago, IL, USA). The data's normality was tested using Shapiro-Wilk's test. Normally distributed data were reported as mean ± standard deviation whereas not normally distributed data were reported as medians with interquartile ranges. Differences in means were assessed by Student's *t*-test. For differences in medians and interquartile ranges, the Mann-Whitney test was performed. Spearman rho coefficient was used to correlate between variables in the study groups. Correlation analyses were adjusted for multivariate effects using the Benjamini-Hochberg false discovery rate method. The missing data in MBL was accounted for via pairwise deletion. A probability of less than 0.05 was regarded as significant.

## 3. Results

### 3.1. Demographic, Medical, and Clinical Characteristics

The characteristics for periodontitis patients and controls are shown in Table 1. Amongst the periodontitis group, seven were diagnosed diabetics whereas five were undiagnosed at the time of study inclusion. Periodontal disease severity parameters and number of sites affected were significantly increased in periodontitis patients. Glycated proteins HbA1c and FrAm were also increased in periodontitis patients. WC was increased whereas BMI was comparable.

### 3.2. Association with Periodontal Disease and Anthropometric Parameters

The results for association of HbA1c and FrAm with periodontal parameters, BMI, and WC are shown in Table 3. HbA1c showed moderately strong associations with all parameters except for BMI and *Σ*MBL. FrAm showed a strong association with missing teeth. MBL levels were directly associated with both glycated proteins.

### 3.3. Correlation of HbA1c with Fructosamine

**Figure 2:**
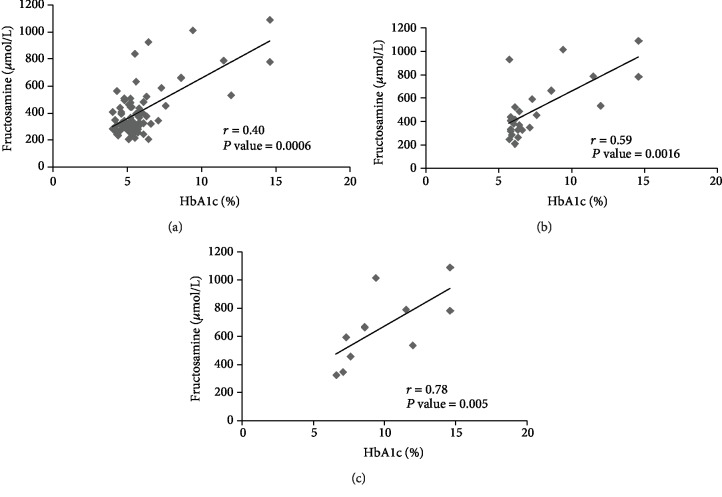
Scatter plots showing correlation coefficients between fructosamine and HbA1c in (a) all study subjects (*n* = 71), (b) prediabetic and diabetic periodontitis patients (*n* = 26), and (c) diabetic periodontitis patients only (*n* = 12). Spearman rho coefficient *r* was calculated to determine the correlation.

A correlation analyses for HbA1c and FrAm were carried out for all subjects (*n* = 71), prediabetes and diabetes (*n* = 26), and only diabetes (*n* = 12) patients. The strength of the association between the two variables increased across the groups with the strongest association found in diabetics. The results are shown in Figure 2.

### 3.4. Glycated Proteins Associated with Inflammatory Proteins

A correlation analyses between glycated proteins and inflammatory proteins were carried out for 50 subjects (periodontitis = 38, controls = 12). The results are presented as a correlation matrix in Figure 1. HbA1c correlated with 60% of the analyzed proteins. Sixteen proteins 4E-BP1, AXIN1, CCL11, CCL19, CCL25, CX3CL1, FGF-21, IL-10, MCP-1, MIP-1 alpha, MMP-1, SIRT2, SLAMF1, ST1A1, STAMPB, and TNFB were found to correlate inversely with both HbA1c and FrAm. Proteins that directly correlated with both HbA1c and FrAm but failed to reach significance were CD40, HGF, IL-18R1, EN-RAGE, CDCP1, and CSF-1. OPG was the only direct correlation with HbA1c reaching significance.

### 3.5. RANKL, OPG, RANKL : OPG Ratio, and Adjusted *Σ*MBL according to HbA1c Levels

**Figure 3:**
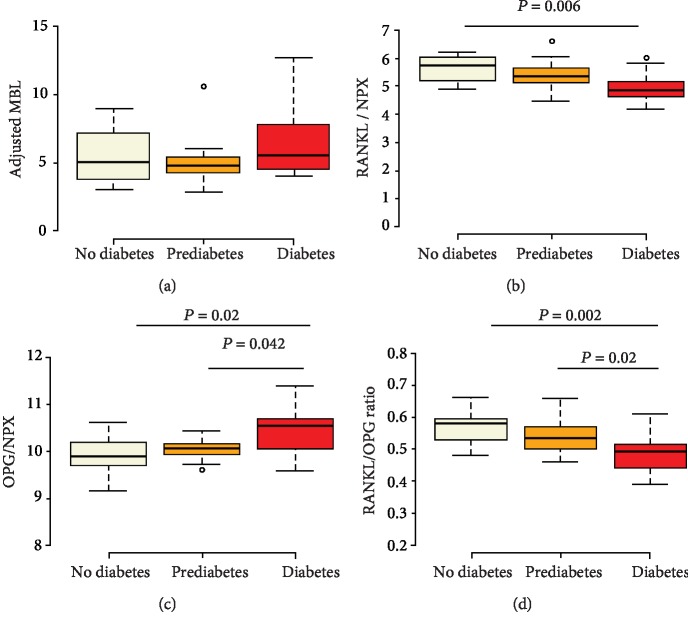
Boxplots showing (a) adjusted MBL, (b) RANKL, (c) OPG, and (d) RANKL : OPG ratio in periodontitis patients (*n* = 38) with no diabetes (*n* = 15), prediabetes (*n* = 12), and diabetes (*n* = 11). Center lines represent the medians; box limits indicate the 25th and 75th percentiles. The whiskers extend 1.5 times the interquartile range from the 25th and 75th percentiles, and the dots represent the outliers. *P* values ≤ 0.05 were considered significant. MBL = marginal bone loss. NPX = normalized protein expression.

For comparison of serum proteins RANKL, OPG, and RANKL : OPG ratio and adjusted *Σ*MBL, periodontitis patients from the PEA analyses (*n* = 38) were divided into three groups based on medical diagnosis and the ADA classification of HbA1c levels of <5.7 (no diabetes), 5.7-<6.5 (prediabetes), and ≥6.5 (diabetes) to determine their bone resorptive status. The results are shown in Figure 3. In the Olink panel, RANKL is referred to as TRANCE (TNF-related activation-induced cytokine). There was no difference in the medians for adjusted *Σ*MBL values between the three groups. However, the serum levels of RANKL were lower and OPG higher in diabetic periodontitis patients as compared to nondiabetic and prediabetics. The RANKL : OPG ratio was the highest in nondiabetic patients as compared to prediabetics and diabetic periodontitis patients.

### 3.6. Analyses of Periodontal Parameters according to HbA1c Levels

**Figure 4:**
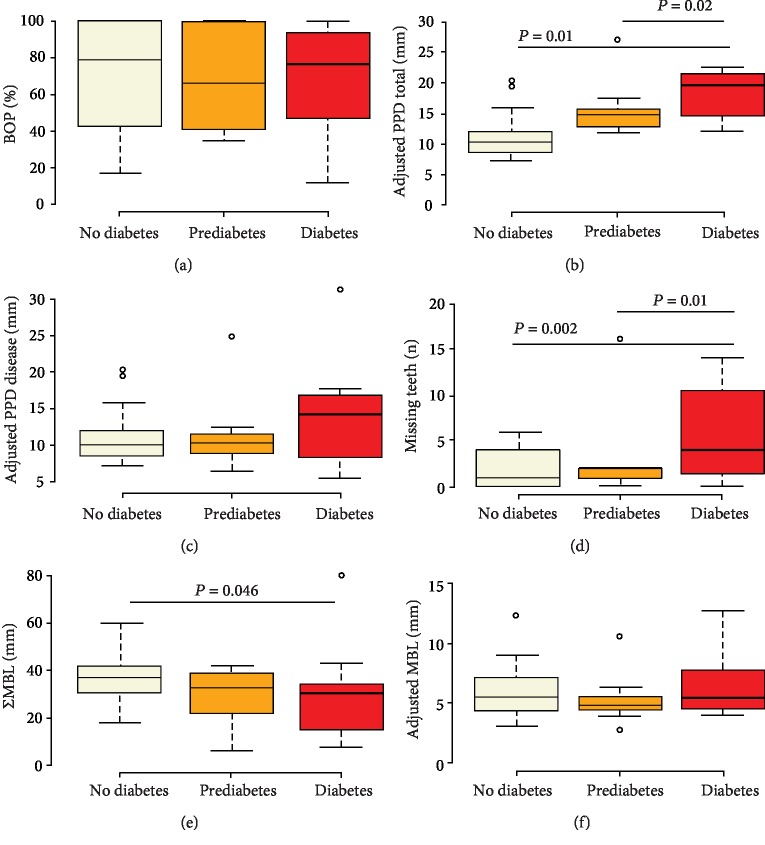
Boxplots showing (a) BOP, (b) adjusted PPD total, (c) adjusted PPD disease, (d) number of missing teeth, (e) *Σ*MBL, and (f) adjusted MBL in periodontitis patients (*n* = 51) with no diabetes (*n* = 25), prediabetes (*n* = 14), and diabetes (*n* = 12). Center lines represent the medians; box limits indicate the 25th and 75th percentiles. The whiskers extend 1.5 times the interquartile range from the 25th and 75th percentiles, and the dots represent the outliers. *P* values ≤ 0.05 were considered significant. PPD = probing pocket depth. MBL = marginal bone loss.

A comparative analysis for periodontal disease severity parameters was performed for all periodontitis patients (*n* = 51) who were grouped into no diabetes, prediabetes, and diabetes according to medical diagnosis and the ADA classification of HbA1c levels. The results are shown in Figure 4. Adjusted PPD total and number of missing teeth were the highest in diabetes as compared to prediabetes and nondiabetes. The medians for BOP and PPD disease were comparable. Regarding *Σ*MBL, nondiabetes patients had higher values compared to diabetes but after tooth adjustment, cumulative bone loss was comparable between all three groups.

## 4. Discussion

The bidirectional relationship between hyperglycemia and periodontitis is supported by numerous studies which depict that periodontal disease severity, and its subsequent part in systemic inflammation can be diabetogenic while sustained hyperglycemia is known to be inimical to periodontal health [15]. Not surprisingly, periodontitis has been described as the sixth complication of type 2 diabetes [16, 17].

Chronic hyperglycemia is a manifestation of insulin resistance which is a pathological condition characterized by insulin's decreasing efficacy at lowering blood glucose levels. Insulin resistance is present throughout prediabetes and type 2 diabetes [17]. The potential role of inflammation is well known in periodontitis. Increasing data also suggest the same for the pathogenesis of type 2 diabetes: that it is immunoinflammatory [18].

This research paper is aimed at looking at the association of glycated proteins HbA1c and FrAm with several proteins of inflammatory interest as well as periodontal disease parameters in periodontitis patients. We found elevated levels of HbA1c and FrAm in patients with periodontitis as compared to controls. A recent study has confirmed our finding of elevated HbA1c values [19]. There is no data available on FrAm levels in periodontitis; however, it has been found to correlate with gingival bleeding in type 2 diabetes patients with periodontal involvement [20].

Regarding measures of glycemia, FrAm and HbA1c represent different glycated targets as the former integrates plasma glucose and their nonenzymatic glycation to plasma proteins and the latter representing the same in an intraerythrocytic compartment. Their reported correlation amongst diabetics is very strong [21]. We report a similar strength in correlation for periodontitis patients with type 2 diabetes. Comparatively, the correlation for the total number of study participants was moderate in strength (*r* = 0.40). There are no studies exploring this correlation specifically in periodontitis patients with or without diabetes; however, a recent study explored this relation in over 20,000 subjects suffering from type 1 or 2 diabetes, prediabetes, or no diabetes. They reported a correlation coefficient of 0.78 in all subjects [22]. The disparity might be due to a much smaller sample size with only 22% type 2 diabetes patients in our study. Also, serum FrAm is significantly influenced by serum protein concentration, particularly albumin which is reportedly lower in periodontitis patients [23].

We also demonstrated the relationship between glycated proteins and periodontal indices, both in terms of disease severity and number of sites affected, in addition to missing teeth. Our results showed a moderately strong association between HbA1c and all periodontal parameters except for *Σ*MBL. Similar results have been reported showing a positive association between poor periodontal health and HbA1c values in nondiabetic subjects [24, 25]. The associations were less consistent for FrAm but nevertheless interesting as it associated with measures of periodontal disease severity. Another noteworthy result is how the number of missing teeth associated with both glycated proteins in similar strength. The only periodontal parameter that has been reported to be significantly correlated with FrAm is gingival bleeding in diabetic patients [26].

To further evaluate the prevalence of periodontal disease severity, we divided the periodontitis group based on the HbA1c glycemic categories of nondiabetes, prediabetes, and diabetes. Disease severity was the highest in diabetic subjects as mirrored by tooth adjusted PPD total and number of missing teeth. The increase in PPD total reflects an increased cumulative of probing depth. This is attributed to periodontal breakdown. In studies on diabetic animals, the connective tissues depict an erratic collagen alignment with high inflammatory cell infiltration [27]. Missing teeth have also been shown to be an important epidemiological marker and risk indicator for type 2 diabetes, cardiovascular disease, and all-cause mortality [28, 29]. HbA1c and WC also showed a positive correlation in our cohort of mainly nondiabetics which has been shown in previous studies as well [30].

The inverse associations of HbA1c and FrAm with inflammatory proteins were a consistent observation, with the exception of OPG. This may be due to the fact that less than a quarter of the sample were patients with diabetes with a BMI in the range of overweight but not obese. This naturally reduces the number of patients exhibiting two established disease states, rather than one. Although, from the clinical associations observed, it seems that chronic inflammation in mostly nondiabetes periodontitis patients seems to affect glycation of serum globulins and hemoglobin to a moderate extent.

We report an important finding here regarding reduced immunoregulatory mechanisms and glycemia under the influence of periodontitis. Both HbA1c and FrAm inversely associated with IL-10 and FGF-21, whereas HbA1c with LAP-TGF-beta and FGF-19, representing a diabetogenic as well as a hyporesponsive immune mechanism.

We have previously shown that T cell aberration and reduced levels of anti-inflammatory IL-10 are associated with severe periodontal conditions [13]. FGF-19 and FGF-21 are late-acting hormones that induce the secretion of insulin and glucagon to regulate glucose homeostasis [31]. Their role in maintaining glucose metabolism has been highlighted by animal studies where the administration of exogenous FGF-19 has been shown to prevent the development of glucose metabolic disorders in mice fed with a high-fat diet [32]. FGF-21 induces lipid oxidation and glycolysis in the liver, also maintaining metabolic homeostasis in other peripheral organs. FGF-21 has a crucial role in the preservation of pancreatic *β*-cells, thus normalizing glycemia [33]. In periodontitis, the underlying immunoinflammatory dysregulation shows a decrease in the capacity of normalizing glycemia, as shown by an inverse relation to FGF-19 and FGF-21.

Both interlerleukin-10 (IL-10) and transforming growth factor-*β* (TGF-*β*) are known anti-inflammatory cytokines secreted by regulatory T cells. IL-10 dampens the cascade of proinflammatory cytokines and downregulates T cell-mediated immune responses [34]. The latency-associated peptide (LAP) is bound as a proprotein rendering TGF-*β* as inactive. The resulting LAP/TGF-*β* complex is highly expressed in regulatory T cells. T cells, dendritic cells, and other immune cells are negatively regulated by TGF-*β* making it immunosuppressive [35]. There are studies showing increased TGF-beta plasma levels in diabetics; however, these have been pinned to a specific organ response, that of the kidneys, to hyperglycemia indicating nephropathy [36]. Regarding IL-10, there are in vitro studies demonstrating their reduced anti-inflammatory function in hyperglycemic conditions which affect IL-10 signaling [37].

In agreement with our findings, elevated levels of serum OPG have been reported in patients affected by type 2 diabetes [38]. The increased OPG levels are attributed to inflammation-driven hyperglycemia and not just high glucose levels per se. In concomitance with increased OPG, decreased levels of RANKL have been reported in diabetic patients with respect to normal subjects [39]. We also found decreased RANKL levels in diabetic periodontitis patients. Furthermore, we also determined the bone resorptive status by calculating the RANKL/OPG ratio. Our results showed that nondiabetic periodontitis patients had a higher bone resorptive status as compared to those with diabetes. The decreased RANKL levels have been attributed to an increase in bone precursor cells with immature osteoblasts and osteoclasts affecting bone turnover in diabetes [40]. Increasing glucose concentrations are cytotoxic for osteoblasts via induction of apoptosis resulting in reduced bone formation [41]. Not only does incremental growth of bone critically depend on osteoblasts but also their provision of the RANK ligand for osteoclastic binding in bone coupling as well as resorption. Even though RANKL is expressed by immune cells, it is its deficiency in the osteoblast lineage that suppresses bone loss as compared to loss of RANKL in T cells which confers no such protection [42]. Our results indicate that hyperglycemia significantly alters OPG and RANKL levels, thereby altering bone resorptive mechanisms in type 2 diabetes.

## 5. Conclusion

Periodontal inflammation associates strongly with measures of glycemic regulation. The anti-inflammatory and antidiabetic control mechanisms are suppressed in periodontitis patients. Bone resorptive mechanism in periodontitis is subdued in the presence of hyperglycemia.

## Figures and Tables

**Table 1:** Demographic, medical, and clinical characteristics in periodontitis and control groups. Data are presented as mean ± SD, median ± interquartile range, or as *n* (%).

Characteristics	Periodontitis	Control	*P* value
*n*	51	20	
Age (years), mean ± SD	47 ± 9.5	43 ± 6.3	
Females, *n* (%)	33 (64.7)	8 (40)	
Smokers, *n* (%)			
Current	6 (11.8)	2 (10)	
Never	45 (88.2)	18 (90)	
Medical factors			
Basic metabolic index (kg/m^2^), mean ± SD	25.2 ± 3.1	24.1 ± 3.4	0.21
Waist circumference (cm), mean ± SD	108.4 ± 14.7	88 ± 14.7	<0.00001
Diabetes mellitus, *n* (%)	12 (23.5)	0	—
Diagnosed	7 (58.3)	0	—
Undiagnosed	5 (41.7)	0	—
Medication, *n* (%)			—
Metformin Sulfonylurea Metformin+sulfonylurea Insulin	3 (42.9) 7 (100) 3 (42.9) 0 (0)	—	—
Periodontal disease severity			
BOP (%), median ± IQR	69.9 ± 28.9	26.1 ± 26.8	<0.00001
*Σ*PPD total (mm), mean ± SD	395.4 ± 74.5	194.9 ± 17.9	<0.00001
Adjusted PPD total, median ± IQR	15.5 ± 3.85	6.9 ± 0.95	<0.00001
*Σ*PPD disease (mm), mean ± SD	237.7 ± 102.9	2.3 ± 3	<0.00001
Adjusted PPD disease, median ± IQR	10.4 ± 3.95	NA	
*Σ*MBL, median ± IQR	33.1 ± 18.6	8.8 ± 16.3	<0.00001
MBL, median ± IQR	5.1 ± 1.8	2.9 ± 0.8	<0.00001
Missing teeth, median ± IQR	2 ± 4	0 ± 0	0.0018
Number of sites affected, *n*			
PPD 3-<5 mm, mean ± SD	44.1 ± 17.9	27.3 ± 14.4	<0.001
PPD ≥ 5 mm, mean ± SD	34.8 ± 14.6	0.4 ± 0.5	<0.00001
Glycated biomarkers			
HbA1c % (mmol/mol), median ± IQR	5.7 ± 1.2 (38.5 ± 12)	4.4 ± 0.6 (25 ± 7.5)	<0.00001
Fructosamine (*μ*mol/L), median ± IQR	380 ± 201	295 ± 94	0.0083

BOP = bleeding on probing; PPD = probing pocket depth; MBL = marginal bone loss.

**Table 2:** ADA's diagnostic criteria used for the diagnosis of type 2 diabetes based on HbA1c values.

	Normal glucose tolerance	Prediabetes	Type 2 diabetes
HbA1c	≤5.7% (39 mmol/mol)	5.7%-6.4% (39-46 mmol/mol)	≥6.5% (48 mmol/mol)

ADA = American Diabetes Association.

**Table 3:** Correlation of glycated proteins with periodontal and anthropometric parameters in all study subjects (*n* = 71). Spearman rho coefficient was used to calculate *r* between two variables. *P* values < 0.05 were considered significant.

Parameters	Fructosamine		HbA1c	
	*r*	*P* value	*r*	*P* value
BOP (%)	0.22	0.06	0.35	<0.01
3-<5 mm (*n*)	0.14	0.25	0.25	<0.05
≥5 mm pockets (*n*)	0.13	0.28	0.43	<0.001
*Σ*PPD total (mm)	0.19	0.10	0.49	<0.0001
*Σ*PPD disease (mm)	0.29	<0.05	0.51	<0.0001
Tooth adjusted PPD total	0.33	<0.01	0.56	<0.0001
Teeth with PPD disease	0.13	0.29	0.40	<0.001
Missing teeth (*n*)	0.41	<0.001	0.48	<0.0001
MBL (mm)	0.27	0.02	0.45	<0.0001
*Σ*MBL (mm)	0.01	0.94	0.18	0.14
BMI (kg/m^2^)	0.08	0.50	0.13	0.29
WC (cm)	0.19	0.11	0.41	<0.001

BOP = bleeding on probing; PPD = probing pocket depth; MBL = marginal bone loss; BMI = basic metabolic index; WC = waist circumference.

## Data Availability

The data used to support the findings of this study are included within the supplementary information files.

## Supplementary Materials

Supplementary 1 Data S1: data on age, BOP, PPD 3-<5 mm, PPD = 5 mm, SPPD total, SPPD disease, missing teeth, tooth adjusted PPD total, tooth adjusted PPD disease, MBL, SMBL, adjusted SMBL, HbA1c %, HbA1c mmol/mol, fructosamine, BMI, and WC for total subjects (*n* = 71).

Supplementary 2 Data S2: data on serum levels of 70 cytokines from the Olink inflammation analyses for study subjects (*n* = 50).
