# Supplementary material for: Association of Glycated Proteins with Inflammatory Proteins and Periodontal Disease Parameters
Source: J Diabetes Res. 2020 Jan 10;2020:6450742. doi: 10.1155/2020/6450742 (PMC6977320; doi:10.1155/2020/6450742)
Supplement: Supplementary 1 — Data S1: data on age, BOP, PPD 3-<5 mm, PPD = 5 mm, SPPD total, SPPD disease, missing teeth, tooth adjusted PPD total, tooth adjusted PPD disease, MBL, SMBL, adjusted SMBL, HbA1c %, HbA1c mmol/mol, fructosamine, BMI, and WC for total subjects (n = 71). [file 6450742.f1.pdf]

Subject #	Olink analyses	Age	BOP	3-<5mm (n)	≥5mm (n)	ΣPPD TOTAL	ΣPPD DISEA	Missing teeth	Tooth adjuste	Tooth adjust	MBL	ΣMBL	Adjusted ΣM	HbA1C %	HbA1C mmol/r	Fructoseamine	BMI	WC (cm)
P1	Yes	57	100	50	17	368	197	9	19.4	13.1	NA	NA	NA	9.4	79	1014	17.4	81
P2	Yes	53	24	51	29	455	344	5	19.8	17.2	4.9	29.4	4.90	7.1	54	347	28.1	116
P3	Yes	55	95	78	15	418	164	0	14.9	9.6	5.5	44.0	5.50	5.5	37	259	22.2	91
P4	Yes	46	38	15	32	325	297	16	27.1	24.8	10.9	43.5	10.58	5.8	40	440	30.1	114
P5	Yes	52	91	31	32	362	285	12	22.6	17.8	7.6	15.3	7.63	8.6	70	666	31.2	112
P6	Yes	51	79	62	25	466	270	0	16.6	12.9	5.9	35.6	7.12	4.8	29	510	23.1	102
P7	Yes	55	92	40	29	406	297	9	21.4	16.5	10.1	80.5	10.06	14.6	136	783	28.3	105
P8	Yes	60	97	60	28	467	336	5	20.3	20.3	9.0	35.9	8.98	5.2	33	448	22.3	94
P9	Yes	43	100	64	40	381	212	1	14.1	8.5	3.7	29.2	3.65	4.6	27	328	26.1	108
P10	Yes	32	77	64	31	373	161	0	13.3	8.5	4.5	36.3	4.54	5.2	33	509	25.7	127
P11	Yes	38	83	72	32	384	164	0	13.7	8.6	3.8	30.4	3.80	5.1	32	267	25.7	116
P13	Yes	58	100	57	22	338	116	2	13.0	7.3	2.8	5.5	2.76	6.1	43	485	22.3	105
P12	Yes	45	98	48	52	562	401	2	21.6	15.4	5.5	33.1	5.52	14.6	136	1092	21.3	101
P14	Yes	44	38	16	36	329	189	1	12.2	7.9	4.5	31.6	4.52	7.3	56	591	30.5	147
P15	Yes	55	100	50	26	338	139	2	13.0	8.7	4.6	18.4	4.59	6.3	45	380	25.7	107
P16	Yes	63	78	72	41	403	216	1	14.9	8.6	4.3	34.2	4.28	6.6	49	325	30.5	128
P17	Yes	40	93	54	41	373	219	2	14.3	9.1	3.9	15.5	3.88	6.3	45	524	27.8	133
P18	Yes	42	100	55	37	328	193	5	14.3	10.2	3.5	21.0	3.51	5.1	32	464	27.1	131
P19	Yes	37	92	43	50	435	293	0	15.5	10.5	4.9	39.1	4.89	5.8	40	417	24.1	120
P20	Yes	42	100	30	40	380	229	2	14.6	10.4	5.5	22.2	5.55	5.9	41	317	25.7	128
P21	Yes	44	90	27	55	435	290	1	16.1	11.2	4.4	26.5	4.42	4.6	27	412	25.0	113
P22	Yes	50	35	26	51	416	271	0	14.9	10	4.4	35.6	4.45	6.4	46	208	23.2	108
P23	Yes	40	100	38	48	421	274	2	16.2	11.4	4.5	36.0	4.51	5.7	39	261	24.1	116
P24	Yes	45	76	50	52	463	288	0	16.5	10.3	3.8	30.1	3.76	5.2	33	371	24.3	93
P25	Yes	25	100	41	57	496	335	0	17.7	12	3.1	18.4	3.07	5.3	34	231	22.2	82
P26	Yes	50	73	43	26	351	146	1	13.0	9.7	4.7	32.8	4.10	5.8	40	331	26.2	103
P27	No	32	44	30	43	402	264	2	15.5	12	6.2	31.6	6.32	5.7	39	284	25.9	104
P28	No	50	58	23	50	425	305	2	16.3	11.7	5.7	51.9	6.49	5.4	36	283	24.5	104
P29	No	41	59	28	38	353	220	6	16.0	10.5	5.2	38.3	7.66	5.2	33	277	26.0	107
P30	No	56	100	20	48	363	264	5	15.8	12.6	4.2	18.0	4.49	5.2	33	340	26.8	111
P31	No	36	50	24	34	335	176	0	12.0	9.3	5.1	15.4	12.33	4.6	27	313	28.2	112
P32	No	38	18	33	27	338	145	0	12.1	7.6	4.0	33.9	4.24	5.3	34	331	20.3	82
P33	Yes	50	98	62	49	613	545	0	21.9	19.5	6.6	53.3	6.66	4.5	26	444	25.2	110
P34	Yes	56	75	40	11	243	130	13	16.2	10.8	12.7	12.7	12.70	8.6	70	662	22.6	115
P35	Yes	55	96	9	47	451	437	14	32.2	31.2	7.8	7.8	7.80	11.5	102	789	23.8	97
P36	Yes	32	100	53	33	488	383	4	20.3	15.9	7.5	60.1	7.52	5.6	38	634	20.7	89
P37	Yes	47	12	73	9	361	123	3	14.4	8.2	NA	NA	NA	7.6	60	455	33.1	130
P38	Yes	45	36	53	5	322	143	2	12.4	8.9	5.2	41.9	5.24	5.8	40	370	25.2	112
P39	Yes	40	41	58	22	424	250	1	15.7	10.9	NA	NA	NA	6.4	46	928	26.0	109
P40	Yes	58	43	65	43	408	243	0	14.6	9	5.6	39.3	5.61	5.3	32	445	24.8	126
P41	Yes	56	34	52	26	351	142	1	13.5	8.4	7.7	30.8	7.70	5.6	38	398	27.4	122
P42	Yes	30	100	30	50	452	294	1	16.7	11.3	5.6	44.6	5.58	5.6	38	303	23.5	117
P43	Yes	48	54	62	10	312	50	2	12.0	6.3	5.9	23.8	5.95	6	42	406	27.3	96
P44	Yes	64	56	77	20	389	95	0	13.9	5.6	4.0	28.2	4.03	6.1	43	247	26.6	116
P45	No	60	66	22	65	527	432	2	20.3	16.6	5.3	43.4	5.42	12	108	536	24.1	91.4
P46	No	38	42	28	53	458	321	1	17.0	12.3	6.4	39.7	5.08	5	31	304	27.6	96
P47	No	65	28	19	31	297	162	5	11.9	9	5.1	40.7	5.67	4.8	29	296	23.6	98
P48	No	37	100	53	18	315	94	0	11.3	7.8	4.8	38.9	4.86	4.2	22	287	19.9	76.2
P49	No	43	17	31	11	223	66	4	9.3	7.3	6.9	42.3	6.05	5.5	37	843	25.2	114
P50	No	50	59.8	36	59	489	350	0	17.5	12.5	5.1	41.9	5.24	6.1	43	326	23.0	115
P51	No	47	28	33	27	354	163	0	12.6	8.6	6.9	51.9	7.41	5.4	36	338	22.0	96
H1	No	35	60	62	0	203	n/a	1	7.5	n/a	4.4	8.8	2.93	5.1	32	273	24	118
H2	Yes	49	82	36	1	213	n/a	14	15.2	n/a	5.0	24.9	4.41	4.5	26	271	26.14	118
H3	Yes	60	5	18	0	192	n/a	11	12.0	n/a	3.5	24.3	4.98	4.2	22	351	23.11	106
H4	Yes	44	5	19	1	211	n/a	0	7.5	n/a	3.1	18.3	3.47	4	20	288	30.7	104
H5	Yes	37	7	15	0	224	n/a	0	8.0	n/a	2.3	2.3	3.05	4.3	23	245	21.3	69
H6	Yes	46	14	22	0	186	n/a	0	6.6	n/a	3.3	6.5	2.33	5.2	33	481	24.7	94
H7	Yes	45	91	18	0	195	n/a	0	7.0	n/a	2.6	2.6	3.26	4.8	29	348	23.3	82
H8	Yes	43	9	22	1	197	n/a	0	7.0	n/a	3.0	6.0	2.55	4.4	25	237	18.4	66
H9	Yes	41	15	27	0	183	n/a	0	6.5	n/a	3.1	9.3	3.00	4.3	23	566	19.6	76
H10	Yes	44	0	16	0	208	n/a	1	7.4	n/a	3.0	8.8	2.33	4.8	29	337	23.8	73
H11	Yes	42	28	38	1	201	n/a	0	7.2	n/a	2.9	11.5	2.95	5.5	37	219	22.5	86
H12	Yes	36	34	20	0	175	n/a	0	6.3	n/a	3.2	3.2	2.88	5.6	38	273	22.4	81
H13	No	53	13	48	0	188	n/a	0	6.7	n/a	2.3	2.2	3.20	4.2	22	269	19.3	81
H14	Yes	46	18	16	1	190	n/a	0	6.8	n/a	2.3	2.3	2.23	4.2	22	302	30.1	86
H15	No	42	8	44	0	240	n/a	0	8.6	n/a	2.6	20.0	2.26	4.4	25	265	24.2	92
H16	No	37	13	18	0	168	n/a	0	6.0	n/a	2.4	20.8	2.86	5.1	31	208	26.2	90
H17	No	39	19.6	15	0	185	n/a	0	6.6	n/a	2.8	17.8	2.60	4.3	23	313	26.7	87
H18	No	33	1	8	1	173	n/a	0	6.2	n/a	2.8	20.3	2.97	4	20	408	26	72
H19	No	45	58	38	0	181	n/a	0	6.5	n/a	2.2	8.6	2.54	4.8	29	499	21	84
H20	No	43	41	46	1	184	n/a	0	6.6	n/a	NA	NA	NA	4.6	27	397	29	94
