# Supplementary material for: Association of Glycated Proteins with Inflammatory Proteins and Periodontal Disease Parameters
Source: J Diabetes Res. 2020 Jan 10;2020:6450742. doi: 10.1155/2020/6450742 (PMC6977320; doi:10.1155/2020/6450742)
Supplement: Supplementary 2 — Data S2: data on serum levels of 70 cytokines from the Olink inflammation analyses for study subjects (n = 50). [file 6450742.f2.pdf]

#LOD-Max	2.12	1.06	1.45	0.28	1.33	0.87	0.96	0.85	0.88	0.89	0.28	1.44	0.62
NPX	101_IL-8	102_VEGF-A	105_MCP-3	107_CDCP1	108_CD244	109_IL-7	110_OPG	111_LAP TGF-beta-1	112_uPA	113_IL-6	115_MCP-1	117_CXCL11	118_AXIN1
**P1**	7.54	9.33	NAN	5.69	5.24	1.35	9.97	4.95	9.34	2.91	7.88	NAN	NAN
**P2**	11.50	10.11	3.11	5.11	7.54	4.59	10.38	5.07	10.55	4.42	9.41	1.91	NAN
**P3**	11.69	9.69	1.55	5.38	5.67	2.25	9.90	4.52	9.77	4.07	7.84	NAN	NAN
**P4**	13.08	9.68	NAN	3.53	6.11	3.46	10.05	4.86	10.04	4.77	8.93	1.45	NAN
**P5**	10.82	9.28	NAN	3.83	5.47	1.19	10.72	4.07	9.61	3.38	7.81	NAN	NAN
**P6**	12.43	9.00	3.20	2.92	6.69	4.73	10.12	5.64	10.10	6.33	9.69	2.27	NAN
**P7**	13.38	9.00	1.68	5.74	6.44	2.35	10.69	4.58	10.10	5.98	9.42	2.13	NAN
**P8**	6.38	8.87	NAN	3.18	5.98	2.49	9.16	4.30	9.56	2.94	8.06	NAN	NAN
**P9**	12.26	10.34	NAN	3.27	6.70	4.56	10.61	5.14	10.52	4.94	8.23	2.37	0.68
**P10**	10.71	10.08	1.95	3.21	6.59	4.60	10.41	5.56	10.62	4.71	9.09	5.01	1.14
**P11**	11.66	9.79	NAN	3.02	5.87	2.96	10.28	4.91	10.15	3.39	9.19	1.60	NAN
**P13**	13.27	10.15	4.81	3.51	6.79	4.67	10.06	6.66	10.60	7.21	9.72	7.27	1.33
**P12**	10.82	9.87	NAN	4.74	6.21	2.51	10.65	4.64	10.03	3.86	8.33	NAN	NAN
**P14**	14.13	10.15	2.91	3.69	6.52	3.08	9.58	5.38	10.16	4.82	8.87	1.86	NAN
**P15**	8.31	8.89	1.79	4.17	6.30	3.86	9.73	4.77	10.13	3.45	9.98	1.99	0.69
**P16**	14.22	10.68	NAN	3.21	6.10	2.55	11.39	4.57	9.98	7.38	9.78	NAN	NAN
**P17**	10.01	10.26	2.74	3.45	6.53	4.75	9.93	5.32	10.12	4.29	9.90	5.01	0.99
**P18**	14.75	10.54	10.69	4.61	6.99	4.54	9.88	6.14	10.04	10.20	12.91	2.31	NAN
**P19**	11.74	10.47	2.22	3.17	6.73	4.14	10.40	5.30	9.58	4.30	8.45	3.10	1.31
**P20**	14.75	9.36	1.71	3.36	6.59	3.77	9.62	5.17	10.10	6.73	9.58	2.52	NAN
**P21**	7.59	9.30	NAN	3.27	6.56	3.94	9.40	4.82	10.07	3.47	8.57	NAN	NAN
**P22**	14.18	10.32	4.67	2.71	6.31	3.64	10.05	4.78	10.28	4.08	9.03	1.73	1.64
**P23**	14.95	10.40	6.37	3.49	7.07	5.18	10.44	5.19	10.83	8.65	9.66	3.09	1.90
**P24**	14.17	10.49	4.24	4.24	6.39	3.86	9.74	6.79	10.59	6.17	10.26	7.58	2.78
**P25**	14.87	9.89	5.25	2.54	6.82	4.44	9.72	5.90	10.72	8.99	9.65	3.74	2.03
**P26**	15.09	10.50	7.14	3.30	6.89	4.51	10.11	5.71	10.70	8.16	10.47	7.22	2.84
**P27**	14.73	10.46	1.77	3.99	5.48	1.44	9.70	4.16	9.90	7.65	9.80	2.70	NAN
**P28**	7.51	9.48	1.67	3.28	5.95	1.85	10.06	4.64	9.66	2.93	8.32	NAN	NAN
**P29**	8.37	8.25	NAN	4.69	6.10	1.73	10.56	4.46	9.57	3.51	6.94	NAN	NAN
**P30**	9.34	9.56	1.74	4.06	6.11	5.27	10.05	5.51	10.22	5.41	9.60	2.12	NAN
**P31**	14.72	10.40	2.33	4.13	5.22	2.60	10.07	4.75	10.28	5.20	8.36	NAN	NAN
**P32**	14.51	9.83	2.49	3.74	4.34	1.18	10.10	3.73	9.70	5.42	8.42	1.67	NAN
**P33**	14.35	9.96	5.89	3.78	6.46	5.16	10.20	5.39	10.12	7.93	9.63	3.67	1.00
**P34**	11.27	10.19	3.12	3.49	6.31	4.40	9.37	6.00	9.36	2.49	8.92	1.63	NAN
**P35**	14.69	11.80	11.71	4.40	6.99	3.18	9.93	5.53	10.96	11.12	12.89	6.31	1.56
**P36**	14.90	11.28	8.97	3.75	7.90	4.68	10.38	6.99	10.92	7.32	10.48	4.87	1.71
**P37**	14.72	9.99	2.12	3.75	5.65	1.72	9.96	4.16	10.20	7.21	10.07	NAN	NAN
**P38**	14.46	11.05	10.48	3.58	6.83	4.91	10.69	5.06	10.51	12.86	12.59	1.83	0.62
**H1**	12.96	9.73	1.49	2.81	5.40	1.26	9.58	4.26	9.22	3.51	7.66	2.50	NAN
**H2**	14.83	9.51	5.77	4.64	6.60	3.93	10.70	5.06	9.97	6.87	10.85	1.69	NAN
**H3**	13.15	11.43	4.49	3.50	6.65	5.33	9.95	7.12	10.21	6.40	10.14	7.66	2.29
**H4**	13.20	10.35	5.61	3.33	6.79	5.44	9.60	7.83	10.26	5.36	10.87	7.04	2.75
**H5**	13.30	10.93	4.19	3.75	6.46	5.15	9.80	7.35	10.30	6.99	10.75	7.51	2.42
**H6**	12.13	11.05	3.45	3.15	7.05	5.52	9.90	8.04	10.47	5.52	10.31	7.94	3.18
**H7**	13.46	10.20	4.74	2.77	6.88	4.56	9.81	7.25	10.37	4.20	9.82	7.96	2.71
**H8**	13.14	10.64	4.85	2.46	6.57	4.96	9.85	7.20	10.32	3.69	10.12	6.98	2.46
**H9**	12.43	11.42	4.80	3.93	7.08	5.39	9.83	7.65	10.83	4.22	10.11	7.83	3.42
**H10**	12.95	10.12	4.10	2.68	6.39	5.30	9.51	7.60	10.22	4.10	10.47	7.78	3.30
**H11**	13.62	11.19	6.41	3.09	6.50	5.15	9.76	7.27	10.43	4.39	10.39	7.27	2.96
**H12**	12.98	10.90	4.53	3.13	7.16	5.75	10.15	8.09	10.55	4.53	9.88	7.09	3.29

0.42	1.32	0.37	-0.22	0.89	-0.13	-0.28	0.62	-0.19	1.47	-1.65	-0.20	1.21	-0.09
120_TRAIL	122_CXCL9	123_CST5	126_OSM	128_CXCL1	130_CCL4	131_CD6	132_SCF	133_IL-18	134_SLAMF1	135_TGFA	136_MCP-4	137_CCL11	138_TNFSF14
4.72	3.11	6.28	4.51	1.93	1.70	3.94	2.83	6.37	NAN	-1.33	NAN	NAN	4.81
7.39	7.57	6.34	6.06	5.34	5.54	4.57	5.38	8.50	3.62	2.92	2.56	2.52	6.32
5.25	6.16	5.88	4.77	6.26	2.79	3.54	3.22	5.73	NAN	2.01	0.11	NAN	5.39
7.02	7.45	5.94	6.34	5.81	5.19	3.73	3.71	6.97	1.82	2.64	1.88	NAN	5.71
6.37	5.62	5.80	4.52	3.25	0.25	3.21	1.60	7.00	2.24	2.35	-0.06	NAN	4.87
7.40	7.92	6.29	5.54	7.69	7.11	3.63	5.01	6.79	2.33	2.54	3.66	1.84	5.47
5.95	8.11	6.53	6.09	6.56	2.95	4.57	2.63	8.32	2.69	3.70	0.53	NAN	5.63
6.40	6.51	6.64	3.90	5.51	-0.06	3.88	3.18	5.90	1.91	0.66	0.32	NAN	4.29
7.02	7.66	6.60	5.00	9.72	5.41	3.87	3.42	7.84	2.71	3.11	1.43	NAN	5.93
6.64	7.88	7.44	5.86	8.83	5.39	3.62	5.50	7.15	3.38	3.57	3.84	3.47	6.53
7.09	6.95	6.13	4.84	2.73	2.21	3.96	2.82	6.38	1.50	2.97	1.80	3.93	6.64
7.51	8.67	6.49	6.17	8.27	8.52	4.10	7.81	7.72	3.43	3.48	3.48	5.52	6.58
6.86	6.43	6.10	5.35	3.39	5.47	3.89	2.13	7.21	2.40	3.50	1.37	NAN	5.09
6.73	7.78	6.33	6.59	6.61	6.30	4.36	3.09	6.54	2.35	3.47	2.71	1.26	6.37
6.42	7.84	6.21	3.93	6.06	4.07	3.55	5.23	6.79	2.40	2.12	1.70	NAN	4.89
6.41	7.41	6.78	5.49	3.58	2.57	3.97	3.59	7.10	2.18	3.06	2.87	1.28	6.18
7.16	9.93	6.41	5.60	8.68	2.97	3.89	6.19	7.05	3.04	3.49	4.52	4.01	6.06
7.18	7.10	5.54	8.44	7.67	2.67	4.28	4.00	7.03	2.68	2.84	1.72	1.35	6.58
6.51	8.34	5.92	5.49	5.62	5.65	4.02	5.30	6.78	2.81	3.33	2.02	2.74	5.53
7.22	9.20	6.34	6.94	9.63	3.09	3.89	3.67	7.11	2.97	3.33	2.58	1.21	6.16
6.54	6.94	6.87	4.39	6.45	0.51	4.21	3.81	6.13	2.22	1.17	1.37	NAN	5.03
6.77	7.24	6.26	6.73	6.83	7.22	3.97	6.36	6.69	2.29	3.50	2.62	3.26	5.80
7.36	7.36	6.37	7.14	7.19	7.92	4.62	5.21	6.36	2.79	4.43	2.53	3.32	6.89
7.43	8.47	6.77	7.13	9.19	7.27	4.72	9.93	7.59	3.42	3.96	4.36	8.33	7.38
6.68	7.13	6.45	7.17	8.29	8.80	3.82	6.87	6.64	2.87	3.71	3.32	4.79	6.87
6.95	7.71	6.73	8.20	9.57	6.86	4.42	7.30	7.29	3.15	3.82	3.60	4.98	7.10
7.08	9.92	6.16	7.26	2.91	3.81	3.99	2.78	7.26	1.59	3.54	-0.12	NAN	6.19
6.38	6.61	6.39	2.93	2.64	3.00	4.16	4.03	6.62	2.19	1.50	0.14	NAN	4.42
6.30	7.09	6.66	4.46	4.28	2.69	3.96	3.77	6.93	2.09	0.93	0.02	NAN	5.32
6.80	8.91	6.65	4.80	7.01	1.42	3.22	3.56	7.11	2.97	2.45	3.57	1.63	5.48
7.50	5.35	6.09	7.33	4.30	5.23	3.70	5.13	6.91	2.53	4.38	0.00	NAN	6.23
6.65	4.49	5.58	6.15	3.57	4.26	3.54	3.97	6.68	2.35	2.25	NAN	NAN	4.58
6.59	7.56	6.25	6.00	8.74	5.10	3.64	5.14	6.99	2.72	2.79	4.28	3.40	6.42
6.19	6.81	6.59	5.05	2.35	5.00	3.32	5.53	6.26	2.46	2.39	1.79	2.28	5.03
6.25	8.75	8.90	8.29	10.53	9.51	5.06	7.26	8.13	4.00	4.40	3.46	4.84	7.53
8.19	9.12	6.57	7.97	8.32	8.97	5.36	5.81	8.31	3.76	4.99	3.95	4.10	6.94
6.16	6.21	6.57	7.63	6.75	6.94	3.53	5.17	8.30	2.01	2.05	0.00	NAN	5.98
7.23	7.26	8.72	8.38	10.37	10.28	3.71	6.56	7.30	3.52	2.91	3.01	3.44	6.26
6.55	5.45	5.43	5.90	2.55	2.79	3.29	4.36	7.08	2.59	2.23	-0.01	NAN	4.84
6.22	7.32	7.50	6.89	5.51	6.81	3.58	4.97	6.93	2.49	2.63	1.27	1.87	5.73
7.57	7.80	6.99	6.89	9.58	8.38	4.28	8.84	7.52	3.66	4.72	2.91	7.45	7.26
7.62	8.00	6.09	6.30	9.39	7.59	3.55	9.11	7.54	3.74	4.14	3.49	8.10	6.60
7.58	8.20	6.85	6.61	9.73	7.66	4.02	9.22	8.06	3.10	3.39	3.24	8.26	6.61
8.64	7.77	7.12	6.74	11.02	8.53	4.28	9.48	8.33	3.50	4.67	3.60	8.25	7.27
8.05	7.51	6.63	6.75	9.40	7.12	4.08	9.72	7.34	3.36	3.92	2.91	7.58	6.54
8.07	8.83	7.61	6.57	8.97	6.63	4.12	9.61	7.08	3.47	3.47	3.48	8.65	6.52
8.60	8.57	7.17	6.54	10.24	7.85	4.87	9.72	8.45	3.21	3.44	3.75	9.03	6.65
8.03	7.60	6.71	7.48	8.72	9.32	3.54	9.30	7.94	3.54	3.90	2.92	8.41	6.78
8.37	7.58	7.23	6.99	9.60	8.24	4.14	9.25	7.55	3.57	3.88	3.81	8.50	6.64
7.93	7.64	7.64	5.74	9.53	7.28	4.39	9.34	7.79	4.00	3.03	3.52	9.23	5.62

0.82	-0.49	1.27	0.96	0.99	0.63	1.21	0.71	1.83	0.73	1.44	1.07	0.50	0.27
140_IL-10RA	142_MMP-1	143_LIF-R	144_FGF-21	145_CCL19	148_IL-15RA	149_IL-10RB	151_IL-18R1	152_PD-L1	153_Beta-NGF	154_CXCL5	155_TRANCE	156_HGF	157_IL-12B
NAN	3.56	1.32	NAN	NAN	1.79	3.05	8.74	2.38	1.54	2.49	5.01	9.18	4.62
NAN	4.41	3.34	NAN	2.00	1.39	7.53	9.13	5.11	2.58	4.76	4.51	9.20	5.24
NAN	3.47	1.39	NAN	NAN	1.91	2.24	8.11	3.29	1.58	1.94	4.91	8.97	4.76
0.84	3.32	2.93	NAN	NAN	1.23	4.98	8.04	4.34	1.73	3.89	6.60	9.11	4.68
0.87	2.43	3.50	1.69	NAN	1.19	3.70	8.20	4.53	2.01	4.01	4.45	8.82	2.83
NAN	4.69	3.15	1.36	1.14	1.12	7.39	7.89	5.41	1.95	10.74	5.45	8.64	4.25
1.88	4.04	4.00	1.20	3.62	1.55	4.16	10.10	4.91	2.64	4.92	4.85	9.64	4.41
NAN	4.68	3.26	NAN	NAN	0.91	3.87	8.11	3.94	1.67	3.33	6.00	8.36	3.95
1.32	3.91	3.28	NAN	1.91	1.23	6.79	7.75	5.14	2.86	5.02	6.21	9.09	5.02
1.07	8.21	3.78	1.75	2.80	1.29	7.12	8.50	6.05	2.26	12.08	4.97	9.16	4.67
1.41	4.46	3.75	1.10	NAN	0.96	3.66	8.05	5.12	1.93	3.81	6.04	8.87	3.41
0.89	7.34	3.74	2.36	7.96	1.42	7.91	8.76	5.70	1.95	11.30	5.30	9.03	4.28
NAN	5.15	3.98	NAN	NAN	1.24	4.14	8.68	5.58	2.40	3.56	4.78	9.61	3.51
NAN	6.44	3.57	NAN	3.60	1.08	4.95	7.90	5.83	2.12	5.32	5.81	9.07	4.69
NAN	5.08	3.41	1.04	1.35	0.96	6.29	7.66	5.08	1.52	5.79	4.47	7.91	4.07
0.88	5.55	3.52	NAN	1.30	1.31	4.39	8.47	5.50	1.64	4.23	6.00	9.21	4.01
1.11	7.86	3.24	2.08	2.55	1.18	7.23	7.76	5.73	3.00	11.08	5.35	8.62	4.82
0.99	7.41	2.12	NAN	1.02	1.80	7.29	8.78	6.04	1.59	3.47	5.51	9.53	4.77
NAN	6.49	3.65	1.19	2.05	1.24	7.40	8.24	4.35	1.77	5.51	5.14	8.85	4.05
0.94	5.27	3.18	NAN	1.49	1.10	5.44	7.79	4.91	2.01	3.85	5.49	9.05	4.35
NAN	5.29	2.84	NAN	NAN	1.00	5.58	8.27	3.87	1.72	4.32	6.18	8.42	4.20
1.30	4.45	3.32	1.45	1.04	1.50	7.38	7.87	4.33	2.56	9.16	4.85	8.99	4.88
NAN	4.29	3.95	2.04	4.96	1.19	7.73	8.94	4.40	2.79	7.04	5.42	9.63	4.02
1.05	7.18	3.38	4.48	8.34	1.19	7.40	7.87	4.55	1.55	12.28	6.05	8.86	5.04
NAN	6.65	3.55	1.32	3.46	0.96	7.16	7.66	5.47	3.02	11.60	5.75	8.91	5.21
NAN	5.74	3.73	2.92	11.90	1.49	7.65	7.56	5.79	2.91	12.38	5.79	9.41	5.62
1.05	4.25	2.84	NAN	NAN	1.29	3.71	8.45	5.19	1.87	3.29	5.80	9.41	3.35
0.92	2.68	4.00	NAN	1.28	0.94	3.16	8.06	4.22	1.68	3.62	4.87	7.88	3.30
NAN	5.18	3.17	NAN	NAN	1.30	3.90	8.66	4.77	1.96	3.68	5.03	8.89	3.69
1.10	6.23	3.39	1.59	4.18	1.15	5.89	8.15	5.92	2.39	6.45	5.31	8.56	3.88
NAN	3.48	3.32	NAN	NAN	1.02	3.88	8.83	4.95	2.01	2.62	5.31	10.24	2.28
1.03	4.35	3.01	NAN	2.86	1.11	3.64	7.34	4.77	1.38	3.59	5.16	8.82	3.42
0.93	7.49	4.03	2.90	2.82	0.96	7.19	8.53	5.59	2.38	11.36	6.06	8.46	4.28
NAN	5.98	1.87	1.12	1.36	1.80	7.78	8.02	4.66	1.92	2.87	4.94	8.14	4.10
NAN	5.31	3.56	4.92	4.62	2.18	8.35	8.40	7.45	2.37	11.67	5.07	9.49	6.55
0.83	4.44	4.12	1.16	1.57	1.70	7.83	8.26	5.64	2.68	6.36	6.05	9.66	4.92
0.96	4.33	3.31	NAN	NAN	1.15	3.87	8.70	4.78	1.93	2.68	5.34	9.16	3.53
0.93	5.04	3.71	3.25	NAN	2.45	7.96	8.09	5.95	2.57	7.90	4.20	8.85	4.53
NAN	5.20	2.73	NAN	NAN	1.22	2.78	8.10	4.86	1.52	2.31	4.80	9.11	3.27
0.85	4.04	3.26	NAN	NAN	1.41	6.20	7.45	4.67	2.57	4.04	4.51	8.57	4.21
1.13	6.66	3.28	3.94	8.65	1.75	7.64	8.57	5.11	3.19	12.32	6.01	8.85	5.14
1.24	7.50	3.46	2.93	7.07	1.73	7.67	7.66	5.24	2.45	12.76	6.20	8.54	5.34
NAN	6.30	3.29	6.80	8.29	1.71	7.78	8.00	5.46	2.47	12.69	6.31	8.19	4.72
0.82	8.06	3.32	5.73	9.89	1.57	8.16	8.81	5.78	2.28	12.58	6.80	9.32	5.82
1.21	7.00	3.40	5.28	8.40	1.47	7.47	7.89	4.92	2.59	12.33	6.01	8.45	4.91
NAN	6.92	3.63	4.18	8.32	1.58	7.85	7.84	5.24	1.94	11.43	5.35	8.36	4.41
1.10	7.29	3.24	2.50	9.04	1.42	7.57	7.93	4.06	2.41	13.35	6.12	8.65	5.03
1.34	8.18	3.29	3.75	9.13	0.96	7.22	7.15	5.12	1.96	11.77	5.96	8.84	4.12
0.83	6.18	3.63	6.96	9.97	1.48	7.93	7.80	5.25	2.62	12.93	6.76	8.91	5.02
1.06	8.40	3.72	4.58	9.00	1.54	7.53	7.85	5.22	2.27	12.31	6.02	8.16	5.00

0.55	1.07	1.70	0.47	-0.36	-0.18	1.22	0.69	1.37	0.63	1.16	0.11	0.09	0.54	0.88
158_IL-24	161_MMP-10	162_IL-10	164_CCL23	165_CD5	166_MIP-1 alpha	167_Flt3L	168_CXCL6	169_CXCL10	170_4E-BP1	172_SIRT2	173_CCL28	174_DNER	175_EN-RAGE	176_CD40
0.79	9.10	NAN	2.69	4.16	NAN	9.21	1.15	NAN	NAN	NAN	0.34	6.79	7.10	10.67
NAN	7.04	1.96	8.98	4.56	1.37	8.61	3.10	1.72	NAN	1.16	0.48	7.27	8.45	10.54
0.74	8.18	NAN	7.82	3.57	-0.09	8.76	0.71	NAN	NAN	NAN	0.20	6.46	7.31	10.24
NAN	6.67	NAN	8.93	4.23	1.14	8.86	2.12	NAN	NAN	NAN	0.32	6.59	8.02	10.66
NAN	8.25	NAN	8.69	3.47	NAN	8.82	1.09	NAN	NAN	NAN	0.14	6.73	7.81	10.15
NAN	6.91	2.01	8.74	4.21	6.02	8.72	4.69	2.55	NAN	NAN	NAN	7.12	7.48	10.31
NAN	8.41	3.11	7.98	4.56	0.82	9.17	2.56	4.90	0.65	1.49	0.35	7.19	8.15	10.40
0.70	6.67	NAN	8.34	3.84	NAN	9.00	1.71	NAN	NAN	NAN	NAN	7.00	7.01	9.56
0.77	7.95	NAN	9.57	4.49	0.86	8.69	3.68	1.60	NAN	1.58	0.20	6.71	7.84	10.60
NAN	9.02	3.50	9.02	4.16	3.19	8.86	8.10	4.47	1.02	1.19	0.51	6.99	7.33	9.91
NAN	9.06	NAN	8.98	4.04	-0.04	8.94	2.05	NAN	1.04	1.58	NAN	6.84	8.40	10.60
0.57	8.11	2.38	9.84	4.47	7.91	8.78	8.41	7.84	1.26	1.89	1.01	7.13	6.68	10.33
0.75	8.69	NAN	8.74	4.02	-0.04	8.45	2.35	NAN	NAN	1.29	0.25	6.57	8.61	10.74
NAN	8.11	1.94	8.94	4.55	3.44	9.10	3.67	7.20	NAN	1.24	0.71	6.70	8.55	10.21
0.90	7.90	NAN	8.30	4.05	0.72	9.16	2.58	1.68	NAN	NAN	NAN	6.86	6.27	10.13
NAN	9.53	NAN	9.20	4.29	2.01	9.66	2.04	NAN	NAN	NAN	0.39	6.96	8.34	10.99
0.57	7.87	2.56	9.39	4.58	2.47	8.70	7.32	5.37	0.72	1.41	0.24	6.74	7.87	10.00
0.86	9.71	3.47	7.93	4.56	3.87	9.38	2.32	1.60	NAN	1.24	1.00	6.89	8.16	11.11
1.02	6.46	NAN	8.79	4.12	0.57	8.78	2.62	2.70	1.39	2.15	0.16	6.72	7.89	10.72
NAN	9.08	1.97	9.13	4.29	1.22	8.19	2.11	1.88	NAN	1.36	NAN	6.83	8.83	10.57
0.99	7.01	NAN	8.58	4.17	0.20	9.13	2.65	1.38	NAN	NAN	0.30	7.10	7.93	9.70
0.92	7.38	1.77	8.89	4.02	4.44	8.84	4.05	2.34	0.78	2.52	0.22	7.03	7.60	10.14
NAN	7.86	1.71	9.21	4.39	4.27	9.34	3.68	2.34	2.08	3.89	0.51	7.75	8.86	10.97
NAN	9.34	3.45	9.52	4.50	6.94	8.78	8.93	9.69	5.00	4.74	1.80	8.02	7.06	10.16
0.69	7.13	2.75	9.37	4.36	8.07	8.40	6.39	4.99	0.89	3.34	0.17	7.06	7.22	10.01
NAN	6.75	3.70	8.97	4.35	7.11	8.58	7.08	5.71	2.10	4.11	0.70	7.62	8.17	10.25
NAN	8.65	4.20	8.74	3.77	1.19	8.97	1.60	2.45	NAN	1.16	NAN	7.08	8.09	10.86
NAN	7.44	NAN	8.50	3.93	-0.13	9.01	2.01	NAN	0.97	1.28	0.40	6.90	7.87	10.32
NAN	7.92	NAN	8.36	4.01	NAN	8.83	1.11	NAN	NAN	1.40	NAN	6.90	8.32	10.46
0.85	11.38	2.56	9.31	3.99	1.77	9.22	4.86	2.52	0.69	NAN	0.30	7.13	6.87	10.22
NAN	7.46	NAN	6.96	2.99	2.03	7.99	3.05	NAN	NAN	1.96	0.19	6.97	8.88	10.81
NAN	7.95	NAN	7.38	2.99	2.29	8.96	1.71	2.05	NAN	1.16	0.30	7.25	8.39	10.48
0.58	8.29	3.03	9.72	3.86	6.99	8.85	7.51	5.14	1.31	1.25	0.41	7.04	7.37	9.97
0.68	9.14	NAN	9.91	3.69	1.78	8.59	2.21	NAN	1.93	1.84	0.93	7.26	7.09	10.01
1.28	10.23	3.96	10.11	5.62	8.38	10.06	8.08	7.07	2.67	2.72	0.75	7.58	8.55	11.36
0.71	8.80	3.12	10.15	5.40	4.46	8.62	4.78	3.69	2.58	4.39	0.78	7.95	8.85	11.13
NAN	7.57	2.50	8.95	3.65	1.94	8.83	2.44	1.72	0.67	1.56	0.27	7.81	8.35	10.64
NAN	8.50	3.75	9.53	4.96	11.12	9.22	4.56	1.83	NAN	1.94	NAN	6.94	8.50	11.62
NAN	7.59	NAN	9.40	3.03	0.38	9.06	1.45	NAN	NAN	1.58	0.25	6.43	7.63	10.58
NAN	9.23	NAN	9.24	4.02	3.27	9.42	2.41	NAN	2.19	1.86	NAN	6.79	7.30	10.60
0.59	9.49	4.09	9.32	4.00	7.57	8.52	8.84	8.15	4.86	5.78	0.64	7.50	6.32	10.45
1.68	9.11	4.01	9.17	4.42	7.13	8.48	9.11	8.03	3.95	4.96	1.08	7.05	6.75	10.14
NAN	9.53	4.58	9.34	4.69	5.90	9.39	8.93	7.64	6.12	7.02	1.26	7.77	5.84	10.18
0.66	9.36	5.07	9.91	4.88	6.91	9.14	9.18	8.94	6.39	5.13	0.99	7.28	5.84	10.26
1.23	9.38	4.14	8.85	3.93	4.92	8.25	8.73	8.93	5.27	4.80	0.85	7.96	7.57	9.83
NAN	9.73	4.16	9.89	4.50	4.68	9.19	8.98	8.85	4.56	4.28	1.60	7.64	6.47	10.01
1.39	10.84	4.93	9.58	4.74	6.31	9.19	9.44	9.63	2.49	5.94	2.09	7.80	7.00	10.25
NAN	9.56	5.09	8.74	4.08	6.63	8.29	8.14	8.96	3.95	5.71	1.30	7.21	6.57	10.27
NAN	9.98	4.27	9.82	4.56	5.49	9.07	8.80	8.54	4.03	5.10	1.28	7.32	7.35	10.20
0.72	10.86	4.34	9.28	4.23	5.22	9.29	9.59	8.66	7.18	5.98	2.61	7.65	6.04	10.71

0.48	1.15	0.43	0.72	1.40	1.46	0.61	0.19	1.04	0.07	0.96	0.90	0.33	0.15
179_FGF-19	183_MCP-2	184_CASP-8	185_CCL25	186_CX3CL1	187_TNFRSF9	188_NT-3	189_TWEAK	190_CCL20	191_ST1A1	192_STAMPB	194_ADA	195_TNFB	196_CSF-1
1.62	6.37	0.73	0.94	NAN	6.07	2.75	5.01	6.19	NAN	NAN	4.24	NAN	8.38
5.18	7.80	4.04	1.48	4.80	6.51	0.95	6.39	6.82	0.71	0.97	4.23	0.37	8.64
1.48	5.19	0.47	0.81	NAN	6.79	0.61	4.02	4.46	NAN	NAN	2.46	NAN	8.67
3.28	8.38	2.44	0.87	3.93	6.60	NAN	5.20	6.76	NAN	1.50	3.34	NAN	8.43
1.18	5.34	1.32	0.85	3.42	6.27	0.84	4.56	5.92	NAN	1.63	3.39	NAN	8.28
5.80	9.62	3.59	1.58	5.95	6.41	NAN	5.77	7.12	0.53	2.13	4.01	0.56	8.19
1.72	7.91	2.41	1.04	4.63	6.96	0.94	4.96	7.55	NAN	2.33	2.48	0.90	8.81
2.10	6.17	1.19	0.85	4.65	6.04	NAN	4.41	8.82	0.20	1.60	2.69	7.96	8.30
2.40	8.32	3.19	NAN	4.00	6.80	0.71	6.48	6.99	0.41	2.35	4.17	0.45	8.41
5.93	10.45	4.59	1.86	5.58	5.86	NAN	6.46	7.26	0.52	3.43	3.48	3.01	8.08
0.95	7.10	2.58	1.01	4.14	6.12	NAN	5.35	6.40	1.76	2.69	3.73	0.50	8.14
6.14	10.16	6.89	3.10	6.11	6.71	NAN	8.06	7.74	1.72	3.27	4.45	2.57	8.35
2.04	3.24	1.70	NAN	3.45	6.10	1.58	5.00	6.13	NAN	2.09	3.55	NAN	8.11
1.97	7.69	2.81	NAN	4.42	6.32	NAN	5.36	8.71	0.40	2.46	3.30	1.76	8.18
4.55	8.86	2.12	1.34	4.91	6.45	NAN	4.88	5.81	1.73	1.93	3.24	1.42	8.22
2.20	8.52	2.44	1.61	4.02	6.66	0.75	5.51	8.29	3.12	2.04	3.01	0.62	8.59
6.27	9.38	3.97	4.84	5.84	6.36	NAN	6.09	8.63	1.18	3.12	4.08	1.94	8.28
6.72	8.54	3.18	0.82	3.52	6.82	NAN	5.74	10.19	0.96	1.61	4.20	NAN	9.08
4.02	8.77	5.03	2.53	4.42	6.45	0.69	5.91	5.83	0.92	3.16	4.81	1.21	8.31
3.53	9.19	2.61	NAN	3.77	6.26	0.65	5.62	7.83	0.08	2.03	4.40	3.45	8.18
3.20	7.22	1.63	NAN	5.16	6.14	NAN	5.30	9.30	0.52	1.49	2.87	8.11	8.17
5.52	3.98	5.47	2.76	5.73	6.49	0.72	6.04	6.17	0.36	2.76	4.07	1.99	8.31
5.10	8.79	6.09	2.60	5.78	6.40	1.35	6.91	8.68	1.16	3.79	4.95	2.46	8.42
9.29	10.25	6.73	3.56	6.89	6.36	2.61	8.92	6.69	5.25	3.95	5.27	3.64	8.34
7.64	8.28	5.87	2.79	6.55	6.46	1.51	7.53	8.61	1.96	3.44	4.41	3.47	7.99
5.63	9.12	6.55	3.99	6.20	6.87	2.24	7.81	9.07	2.34	4.21	4.80	3.71	8.30
0.77	6.05	1.62	0.96	3.09	6.94	1.08	4.43	6.20	NAN	1.86	3.36	NAN	8.43
2.41	6.37	1.42	1.14	4.68	6.05	0.67	4.53	4.34	0.08	1.79	3.06	0.42	8.06
0.72	5.98	1.31	NAN	3.53	6.22	NAN	4.46	5.22	NAN	1.72	4.10	NAN	8.65
4.99	9.64	2.19	1.25	4.67	6.38	0.76	6.14	7.68	1.02	2.08	3.71	0.96	8.31
1.28	5.06	1.97	1.21	3.27	6.12	NAN	4.78	5.16	NAN	1.87	4.12	NAN	8.49
0.97	3.93	1.41	1.06	1.93	6.43	1.48	4.46	4.21	NAN	2.62	3.94	0.45	8.02
6.28	10.22	4.61	1.97	6.09	5.81	NAN	6.13	8.18	1.23	2.95	4.02	3.16	7.93
6.33	8.71	1.90	1.04	4.61	6.23	NAN	6.50	6.65	0.76	4.09	4.41	NAN	8.67
6.98	12.73	7.09	2.14	7.38	8.68	0.96	7.11	8.88	1.71	4.10	5.42	1.89	8.91
6.56	8.89	7.98	1.79	5.42	7.35	1.80	7.47	7.17	2.53	5.83	6.45	2.37	8.66
1.11	6.26	2.28	1.29	4.07	6.24	1.09	5.29	6.60	NAN	2.51	4.54	NAN	8.34
6.91	8.73	4.95	NAN	6.32	8.02	NAN	5.90	12.39	0.84	2.19	4.34	0.86	8.96
0.75	4.41	1.68	0.86	2.72	6.28	0.67	4.38	3.39	NAN	2.50	3.76	NAN	8.54
2.61	6.96	2.98	NAN	5.94	6.62	0.80	5.52	7.69	NAN	2.29	3.87	NAN	8.25
7.50	10.76	5.05	5.88	6.29	6.69	1.22	8.82	7.14	2.18	4.25	5.26	4.05	8.52
8.39	10.09	5.53	5.79	7.50	6.23	1.72	8.66	6.11	4.02	4.13	5.06	3.68	8.01
7.71	9.68	5.06	7.07	6.95	6.96	1.52	8.97	5.72	3.88	5.54	5.29	3.77	8.30
8.29	10.60	5.49	6.05	6.75	7.13	1.40	9.05	6.41	4.84	3.81	5.00	3.83	8.52
7.72	9.88	4.96	5.92	6.89	6.56	1.41	9.29	4.97	5.14	3.65	5.59	3.53	8.20
9.09	10.51	5.06	6.57	6.54	6.74	2.28	9.11	7.81	5.02	3.33	5.04	3.69	8.18
8.75	11.03	4.75	7.46	6.93	6.95	2.40	9.46	6.88	5.90	3.86	5.22	4.62	8.39
8.70	11.11	4.96	6.33	6.85	6.48	1.41	8.70	9.39	6.12	3.77	4.70	3.89	7.90
7.49	10.18	5.15	5.83	6.70	6.78	1.78	9.32	6.24	6.43	3.57	4.52	3.84	8.32
9.04	9.55	4.66	7.66	6.66	6.80	2.26	9.51	7.38	5.46	4.16	5.19	3.91	7.99
